# Enhancer-Promoter Communication: It’s Not Just About Contact

**DOI:** 10.3389/fmolb.2022.867303

**Published:** 2022-04-19

**Authors:** Annabelle Wurmser, Srinjan Basu

**Affiliations:** ^1^ Wellcome-MRC Cambridge Stem Cell Institute, Department of Biochemistry, University of Cambridge, Cambridge, United Kingdom; ^2^ Department of Physiology, Development, and Neuroscience, University of Cambridge, Cambridge, United Kingdom

**Keywords:** chromatin mobility, enhancer-promoter communication, enhancer-promoter interaction, transcription, stem cell, cell fate and differentiation

## Abstract

*Cis*-regulatory elements such as enhancers can be located even a million base pairs away from their cognate promoter and yet modulate gene transcription. Indeed, the 3D organisation of chromatin enables the establishment of long-range enhancer-promoter communication. The observation of long-range enhancer-promoter chromatin loops at active genes originally led to a model in which enhancers and promoters form physical contacts between each other to control transcription. Yet, recent microscopy data has challenged this prevailing activity-by-contact model of enhancer-promoter communication in transcriptional activation. Live single-cell imaging approaches do not systematically reveal a correlation between enhancer-proximity and transcriptional activation. We therefore discuss the need to move from a static to a dynamic view of enhancer-promoter relationships. We highlight recent studies that not only reveal considerable chromatin movement in specific cell types, but suggest links between chromatin compaction, chromatin movement and transcription. We describe the interplay between enhancer-promoter proximity within the context of biomolecular condensates and the need to understand how condensate microenvironments influence the chromatin binding kinetics of proteins that bind at *cis*-regulatory elements to activate transcription. Finally, given the complex multi-scale interplay between regulatory proteins, enhancer-promoter proximity and movement, we propose the need to integrate information from complementary single-cell next-generation sequencing and live-cell imaging approaches to derive unified 3D theoretical models of enhancer-promoter communication that are ultimately predictive of transcriptional output and cell fate. In time, improved models will shed light on how tissues grow and diseases emerge.

## Introduction

Mammalian development requires the stereotypic establishment of all cell lineages to occur at the right time, the right place, and in the right proportion (Sagner and Briscoe, 2017). Regulation of developmental genes is thus tightly controlled spatiotemporally, with changes in gene regulatory networks underlying cell proliferation or migration, cell-fate specification, and lineage commitment (Andrey and Mundlos, 2017). However, transcription is by its nature heterogeneous, bursty, and regulated by a complex sequence of molecular events that span a wide spatiotemporal scale (Xie and Liu, 2021). *Cis-*regulatory sequences including enhancers (E) interact with transcription factors (TFs) and chromatin regulators within the three-dimensional (3D) chromatin landscape to precisely and dynamically modulate gene expression (Furlong and Levine, 2018). Nevertheless, how cellular diversity and tissue patterns are encoded transcriptionally and arise precisely remains a challenge for modern biology. Indeed, it remains unclear how spatiotemporal transcriptional patterns are modulated by enhancers located kilobases, even megabases, away from the promoters (P) of their target genes. Particularly, enhancers appear to somehow modulate transcriptional burst frequency and cell-type-specific expression, while core promoters seemingly affecting burst size (Suter et al., 2011; Deng et al., 2014; Bartman et al., 2016; Fukaya et al., 2016; Larsson et al., 2019; Otto, 2019; Rodriguez et al., 2019). To address the nature of this E-P communication requires a better understanding of the relationship between the 3D genome and transcription at the single-cell level.

## 3D Genome Organisation: From Bulk to Single-Cell Analysis

Over the last few years, advances in DNA proximity-based technologies, including Hi-C, GAM and SPRITE, have dramatically improved our understanding of the 3D genome, revealing structures that include CTCF/cohesin-driven chromatin loops, topologically associating domains (TADs) and stripes corresponding to interactions between a loop anchor and contiguous genomic regions (Kraft et al., 2019; Hsieh et al., 2020; Krietenstein et al., 2020). Despite these advances, however, it remains unclear how these structures regulate transcriptional output. Part of the challenge stems from technical limitations of DNA proximity-based technologies, which until recently necessitated pooling millions of fixed cells to achieve sufficient resolution. These bulk approaches reveal population averages rather than provide full 3D structures, making it difficult to relate chromatin organisation to transcription. The advent of single-cell DNA proximity-based technologies (scHi-C, scSPRITE), and live super-resolution imaging, finally allows us to probe 3D genome architecture at unprecedented cellular resolution (Vian et al., 2018; Lakadamyali and Cosma, 2020; Brandão et al., 2021). Moreover, multiplexed super-resolution imaging of RNA now permits imaging of the single-cell sub-nuclear spatial organisation of nascent transcription, allowing us to relate chromatin structure to transcription genome-wide (Shah et al., 2018; Su et al., 2020). However, knowledge of chromatin organisation is for the most part derived from fixed-cell approaches, limiting our probing of how chromatin organisation influences transcriptional change, particularly during highly dynamic developmental processes. As a result, elucidating the relationship between genome architecture, transcription and cell-fate transitions in live cells is of critical importance.

## Enhancer-Promoter Proximity can Drive Transcriptional Activation, But is it Enough?

Transcriptional regulation by enhancers is classically believed to be mediated by physical proximity between the promoter and its enhancer(s) (Furlong and Levine, 2018). Indeed, proximity-based methods and others have revealed “contact” areas between enhancers and promoters, stabilised by proteins such as the Mediator complex, and enriched in activating histone modifications and transcription factor (TF) occupancy (Phillips-Cremins et al., 2013; Bunting et al., 2016; Beagrie et al., 2017; Bonev et al., 2017).

To address the functional relevance of E-P genomic distance in transcriptional control, Barinov and colleagues studied the distance of the five endogenous *Drosophila even-skipped* (*eve*) enhancers relative to the promoter in *Drosophila* embryos during stripe patterning (Barinov et al., 2020). They used a multicolor oligopaint imaging approach in fixed embryos, finding a reduction in transcription activation with increased genomic distance. Similarly, others leveraged the jumping properties of a PiggyBac transposon containing a promoter’s cognate enhancer to systematically generate hundreds of pluripotent cell lines with varying E-P distances activating a fluorescent reporter gene measured by flow cytometry and single-molecule RNA FISH in fixed cells (Zuin et al., 2021). E-P contact probabilities decayed rapidly with increasing genomic distance within the TAD, unveiling a non-linear relationship between transcriptional response, and contact probability. The authors suggest rate-limiting regulatory steps may convert transient E-P interactions into longer-lived promoter states at the transcriptional level, although other models are possible. Indeed, genomic distances do not necessarily reflect 3D distances, necessitating the need for complementary approaches.

**FIGURE 1:**
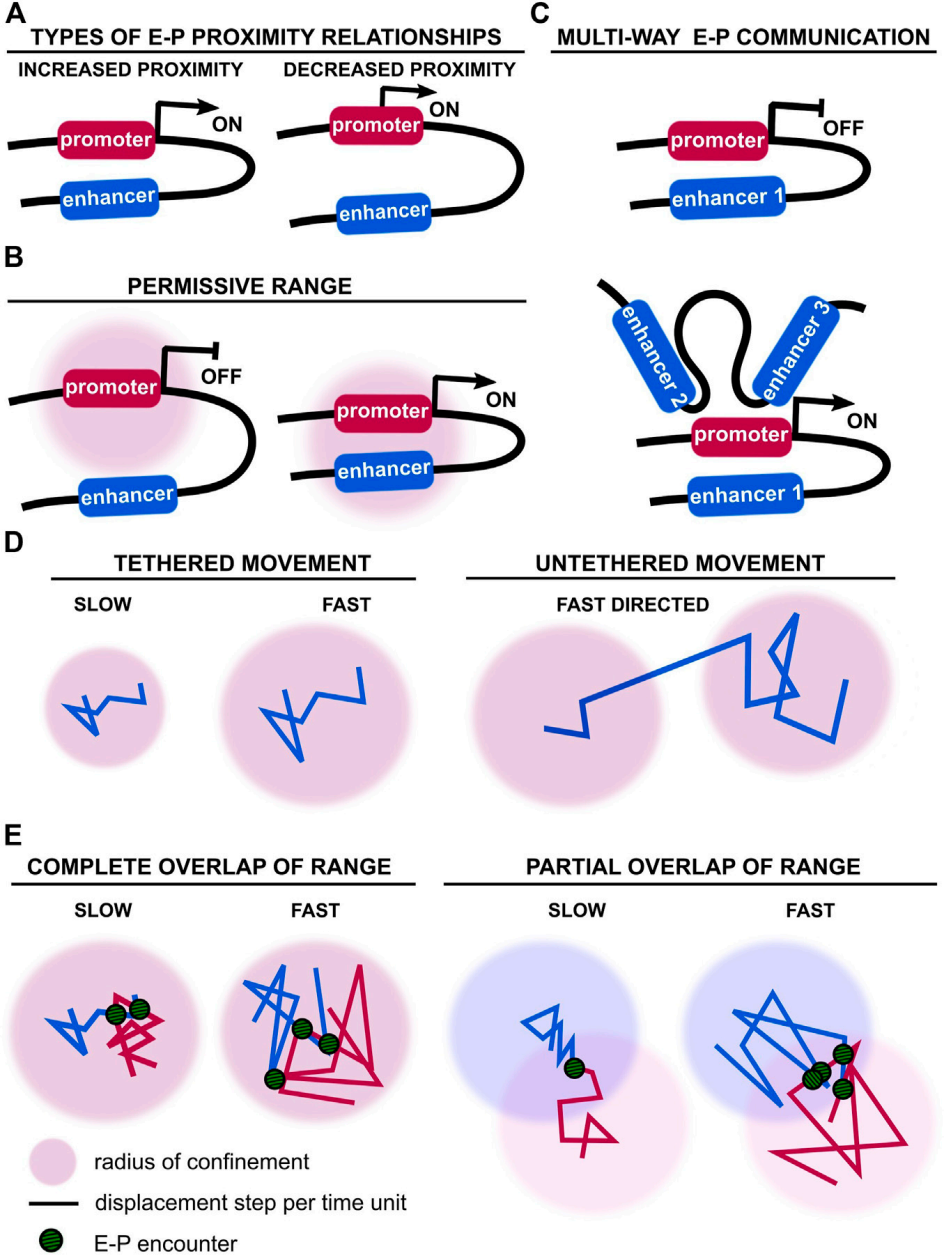
Enhancer-promoter distance and movement vary during transcriptional control. **(A)** Alternative types of observed and theoretical enhancer-promoter distance relationships during transcriptional activation or repression (Chen et al., 2018; Alexander et al., 2019). Different modes of enhancer-promoter activation have been observed for different genes, but also for the same gene in different developmental contexts (Amano et al., 2009; Williamson et al., 2016; Benabdallah et al., 2019). **(B)** Physical engagement of a promoter by its enhancer(s) may not be necessary. Enhancer movement within a permissive range may be sufficient to activate the promoter (Furlong and Levine, 2018). **(C)** Example of a promoter regulated by multiple enhancers. A promoter may not engage with all enhancers simultaneously for activation (Hnisz et al., 2017; Will et al., 2017; Gonen et al., 2018; Osterwalder et al., 2018). **(D)** Alternative types of chromatin movement have been reported, ranging from slow to fast, and existing in tethered and untethered conformations (Khanna et al., 2014; Basu et al., 2020). **(E)** Encounter frequency and duration of E-P communication is dependent on chromatin velocity and respective radii of confinement.

Studies probing the relationship between E-P 3D physical distance and transcriptional activation do not reveal a unified principle (Figure 1A). Support for E-P proximity-driven transcription was first provided when forced looping of the locus control region to the endogenous *β*-globin locus partially rescued *β*-globin expression, a mechanism common to other systems (Deng et al., 2012; Deng et al., 2014; Bartman et al., 2016). Whereas spatial proximity between *Shh* and its limb enhancer (ZRS) was observed in the developing limb (Williamson et al., 2016), spatial separation of *Shh* and its neural enhancers increased during differentiation of mouse pluripotent cells to neural progenitor cells (Benabdallah et al., 2019). Moreover, sequential RNA and DNA FISH revealed only a weak correlation at the *Drosophila* bithorax complex between E-P proximity and nascent transcription (Mateo et al., 2019). No difference was found between active and inactive E-P pairs during early *Drosophila* development using Hi-M, a high-resolution single-cell imaging approach (Espinola et al., 2021). Whether physical proximity is necessary for promoter activation therefore remains an open question.

In addition to these fixed-cell experiments, simultaneous live-imaging of transcriptional activation of a reporter locus by the *eve* enhancers, and E-P distance, revealed that transcription initiation was concomitant with increased E-P proximity in *Drosophila* embryos (distance of ∼340 nm) (Chen et al., 2018). Yet, transcription was not always observed when E and P were close together. In addition, E-P distance was further reduced with sustained transcription, revealing a complex interplay between pre- and post-transcriptional chromatin organisation. However, a live-cell imaging experiment labelling the *Sox2* Control Region and promoter (through targeting of fluorophore-tagged proteins to knock-in arrays of binding sites), and measuring *Sox2* nascent transcription (through targeting of fluorophore-tagged proteins to knock-in MS2 sequences in nascent RNA) showed no correlation between E-P proximity and transcriptional activation in mouse pluripotent cells (Alexander et al., 2019). Instead, E-P distance fluctuated over time and transcriptional bursts were observed even at distances of 600 nm. In contrast, proximity (100–200 nm) of distal enhancer clusters to their target genes *Sox2, Pou5f1,* and *Nanog*, labelled with gRNA-targeted fluorophore-tagged catalytically-dead Cas9 (dCas9), did indeed correlate with nascent transcription monitored by MS2 tagging (Li et al., 2020). Although these findings appear contradictory, they are compatible with enhancer-dependent promoter activation being dependent on E-P distance falling within a *permissive range*, rather than requiring direct physical engagement (Furlong and Levine, 2018) (Figure 1B). The notion of range would reconcile observed differences in E-P distances, ranging broadly from 100 to 300 nm, although E-P proximity, even within a permissive range, is not necessarily *sufficient* for transcriptional activation, as discussed previously.

A more complex relationship between E-P distance and transcriptional activation is illustrated by the important cell-to-cell variability of the *Sox2* locus conformation (Alexander et al., 2019). E-P pairs did not explore the full potential spatial range during time-lapse microscopy, meaning the frequency of observed E-P encounters is highly dependent on the initial locus conformation. It therefore seems unlikely that transcriptional activation is solely mediated by direct E-P contacts; rather, it probably involves additional molecular regulators and physical parameters that may be cell-type, or even gene-locus specific (Fulco et al., 2019). Notably, E-P proximity below a few tens of nm has not been observed, which would be expected in the event of direct physical contact. Whilst this is partly due to technical limitations, e.g. limited spatial resolution of live fluorescence microscopy (Brandão et al., 2021), it may also support models that allow indirect promoter activation. It therefore remains to be shown whether E-P proximity is a consequence or a driving force of further activating steps (Peng and Weber, 2019). Nevertheless, the degree of proximity may increase the probability of transcriptional bursting (Furlong and Levine, 2018).

Finally, E-P communication is rarely one-to-one. Our recent single-cell Hi-C genome-folding structures suggest multi-way E-P relationships, with multiple enhancers and promoters forming intra- or inter-chromosomal 3D clusters that differ from cell to cell (Stevens et al., 2017; Lando et al., 2018) (Figure 1C). Indeed, transcription has been shown to correlate with proximity to the inter-chromosomal region (Cremer et al., 2017; Stevens et al., 2017; Shah et al., 2018). Compounding multiple enhancers increases the number of potential spatiotemporal enhancer combinations to further fine-tune transcriptional control or improve robustness of gene expression, particularly during development. This is exemplified by examples of super-enhancer cooperativity or enhancer redundancy (Bolt and Duboule, 2020).

## Enhancer Movement Correlates With Promoter Activation But Does it Matter and can We Control it?

Beyond proximity, physical parameters such as movement may also provide insights into the functional regulation of enhancers and promoters. However, the relationship between movement and transcription appears to be locus-specific (Ochiai et al., 2015) For example, mean squared displacements and diffusion constants, computed from trajectories of enhancers and promoters labelled using GFP-tagged dCas9, reveal faster diffusion of these *cis*-regulatory elements during transcription, which is (abrogated upon inhibition of transcriptional initiation and elongation) (Gu et al., 2018). This led the authors to propose a “stirring model” whereby accelerated movement increases the encounter frequency of distant enhancers and promoters during transcription. However, if such a mechanism were to provide a positive-feedback loop for transcription, it does not address the role, if any, of E-P proximity for transcription initiation, nor its specificity. Furthermore, real-time tracking of hormone-induced human Cyclin D1 gene expression showed transcription initiation induces gene confinement and altered local diffusion parameters, irrespective of pre-initiation movement (Germier et al., 2017). In addition, single-nucleosome imaging suggests that overall RNAPII locally constrains chromatin during active transcription, but that local chromatin movement differs between transcription initiation and elongation (Nagashima et al., 2019). These studies either suggest that chromatin movement during transcription is locus-specific or that differences somehow arise from the use of alternate imaging methodologies.

Furthermore, our lab and others have uncovered several modes of chromatin movement: slow-diffusing tethered motion, fast-diffusing tethered motion and long-range untethered/directional motion (Basu et al., 2020) (Figure 1D). Slow motion appears to be linked with transcriptional silencing. Out of the two fast-diffusing modes, one is tethered and favours fast exploration of local chromatin, possibly allowing more stable E-P proximity relationships. The other fast-diffusing mode is untethered and corresponds to long-range directed motion, possibly involved in reorganisation of distal E-P proximity relationships. Although fast-diffusing tethered motion has been linked to transcription, less is known about the long-range untethered/directed motion. Initial observations suggest it occurs during transcriptional activation (Levi et al., 2005). Directed motion of a gene locus towards nuclear speckles also significantly increases transcription, although the exact molecular process mediating such direct movement over large genomic distances remains to be unravelled (Khanna et al., 2014). Short- and long-range movement may therefore both influence transcription by bringing enhancers within a permissive range of a cognate promoter on the one hand, and increasing encounter frequencies on the other (Figure 1E).

These studies highlight the importance of identifying molecular mechanisms that control types of chromatin movement, including those that act beyond the transcriptional machinery. For example, chromatin movement may decrease within heterochromatin (Keenen et al., 2021), whereas RNA production may increase long-range movement to form larger heterochromatin domains (Novo et al., 2020). Chromatin decompaction by remodellers and histone acetyltransferases are also likely to control chromatin movement during transcription (Basu et al., 2020; Lakadamyali and Cosma, 2020; Farr et al., 2021). Indeed, local decompaction occurs at both the *Shh* and *eve* genes during transcription (Benabdallah et al., 2019; Barinov et al., 2020). Decompaction itself rather than an increase in chromatin movement may be important for transcription, allowing chromatin binding of proteins required for transcriptional activation (Benabdallah et al., 2019). Furthermore, increased compaction at the *Sox2* locus during neuroectoderm differentiation supports the idea that chromatin compaction mediates changes in transcriptional programmes (Alexander et al., 2019). However, whether compaction represents a general regulatory mechanism, or is locus-specific consequence of transcription remains to be determined (Basu et al., 2020; Yokoshi et al., 2020). In addition to E-P distance distributions, we therefore believe chromatin movement and other biophysical properties may play an important role in instructing transcriptional activation.

## Building Bridges Within Biomolecular Condensates

**FIGURE 2:**
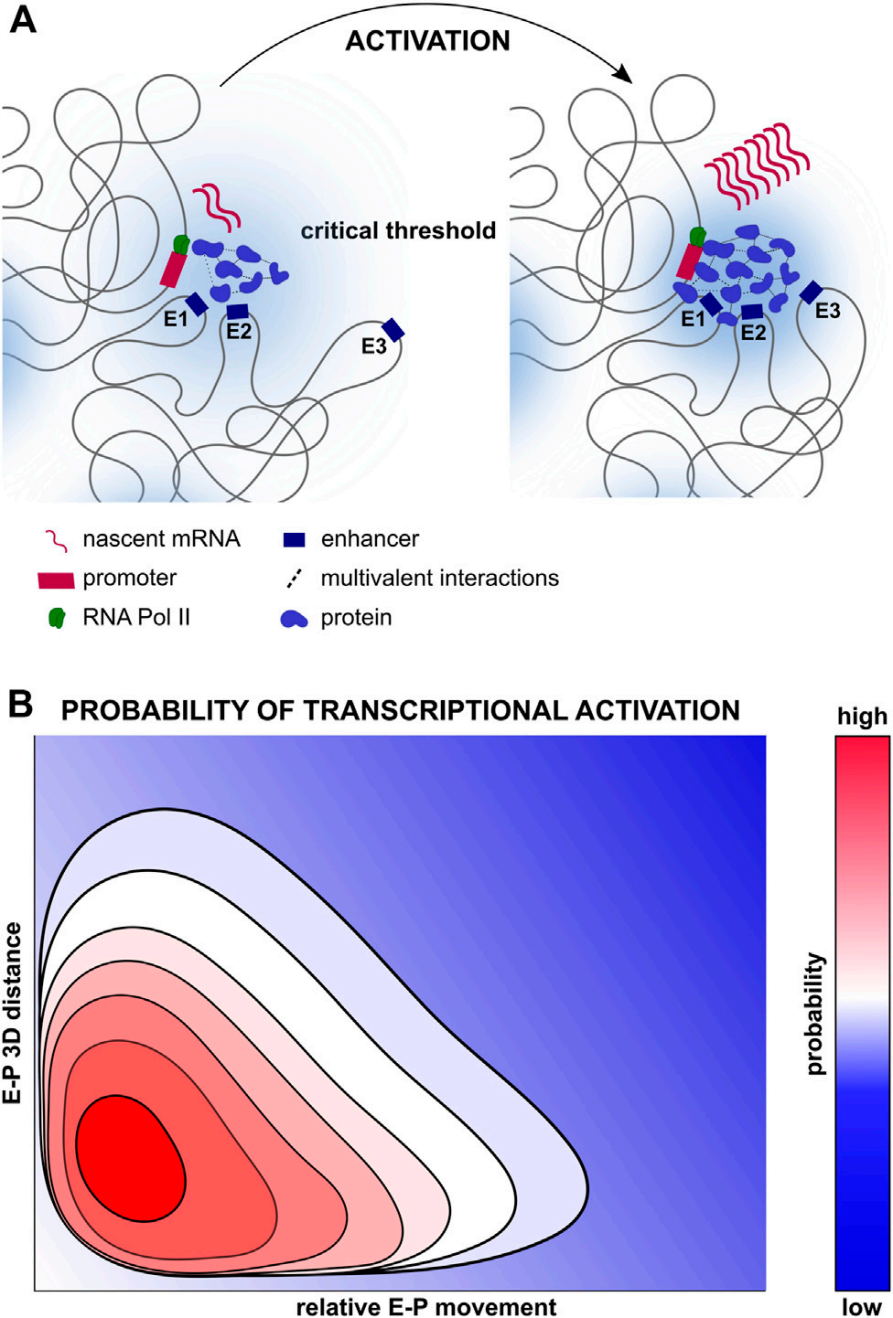
Multifactorial control of transcription. **(A)** Compartmentalisation of transcription regulators, enhancers and promoters all of which may facilitate transcriptional activation. Proteins such as TFs, chromatin regulators, Mediator complexes, RNA polymerases and others may be concentrated in transcription condensates, forming multivalent interactions and increasing local concentrations of transcription regulators, thereby increasing the probability of transcriptional activation (Boija et al., 2018; Cho et al., 2018; Sabari et al., 2018). Depicted on the left is a transcriptionally silent gene locus with few transcription regulators and multivalent interactions, and the presence of only two out of three enhancers within the permissive range. Depicted on the right is a transcriptionally active gene locus with numerous transcription regulators and multivalent interactions, as well as the recruitment of the third enhancer within the permissive range. **(B)** Multidimensional probability distribution of transcriptional activation, ranging from repression (dark blue), to activation (bright red) integrating the numerous biophysical elements regulating transcriptional states spatiotemporally. Increased E-P proximity may increase the probability of transcription activation, but in itself may be insufficient for transcriptional activation, in concordance with the weak, but statistically significant relationship between distance and transcription described in (Xiao et al., 2021). In parallel, greater proximity may increase the frequency of functional E-P encounters, yet proximity in itself may be insufficient to activate transcription (Chen et al., 2018).

Recently, membraneless compartments called biomolecular condensates have gained traction as complementary models for gene regulation (Palacio and Taatjes, 2021) (Figure 2A). They are characterised by elevated concentrations of RNAPII and other transcriptional modulators such as the Mediator complex or TFs (Hnisz et al., 2017; Boija et al., 2018; Shrinivas et al., 2019; Zamudio et al., 2019). Condensates may facilitate both direct and indirect E-P communication, yet ensure robust, specific activation of cognate promoters by nucleating the transcriptional machinery within several hundred nanometers (Heist et al., 2019). Condensates nucleate through recruitment of diffusible molecules by spatially constrained ones (including RNAs, DNA or modified histones), with each condensate exhibiting unique properties with regards to protein density and chromatin/protein mobility (Hnisz et al., 2017; Strom and Brangwynne, 2019; Espinosa et al., 2020; Novo et al., 2020; Welsh et al., 2020; Bhat et al., 2021; Keenen et al., 2021; Saar et al., 2021; Sanchez-Burgos et al., 2021). Such compartments could facilitate establishment of the multi-way regulatory networks between promoters and *cis*-regulatory elements observed during development (Sanyal et al., 2012; Schoenfelder et al., 2015; Freire-Pritchett et al., 2017; Stevens et al., 2017; Novo et al., 2018; Madsen et al., 2020; Chovanec et al., 2021) (Figure 2A).

Moreover, multivalent, low-affinity interactions can result in liquid-liquid phase separation in a concentration-dependent manner, with higher affinity or higher valency interactions sometimes leading to the formation of aggregates or thicker gel-like microenvironments (Khanna et al., 2019; Eshghi et al., 2021). Nevertheless, the necessity of phase separation for transcription activation and elongation remains to be fully established. Indeed, multivalent interactions between TFs seem to increase transcriptional activation capacity through chromatin stabilisation and transcription co-factor recruitment more than phase separation itself (Trojanowski et al., 2021). In addition, transcriptional activation and E-P interactions were uncoupled during Mediator disruption, suggesting Mediator-driven recruitment of enhancers may not mediate the formation of molecular bridges between E-Ps in condensates, although this does not exclude upstream TF-driven clustering (Crump et al., 2021). It is therefore unclear if and how condensates facilitate the spatiotemporal coupling of E-P interactions for transcriptional activation. Another possibility is that condensates simply create microenvironments that allow cells to subtly modulate the kinetics of chromatin binding proteins and chromatin movement, thereby altering the probability of transcriptional activation whilst allowing additional layers of control (Figure 2B). How these subtle changes in transcription subsequently influence mammalian development remains to be fully explored.

## Enhancer-Promoter interactions Could Be Spatiotemporally Decoupled From Transcription

There is growing evidence that establishment of lineage-specific chromatin landscapes may precede changes in gene transcription and cell lineage decisions (Argelaguet et al., 2019). Examples include tissue-specific TF-priming of *Drosophila* mesectoderm enhancers, necessary for synchronised, sustained gene activity, or bookmarking of enhancers during epidermal differentiation (Rubin et al., 2017; Falo-Sanjuan et al., 2019). In addition, *Drosophila* mesoderm enhancers engage in stable chromosomal interactions days before detectable changes in gene expression (Ghavi-Helm et al., 2014; Ing-Simmons et al., 2021). These experiments reveal a complex relationship between local chromatin organisation and transcription, particularly during development. They further stress the need to detect stage and context-dependent regulatory mechanisms at the single-cell level and emphasise the importance of temporal control, as illustrated by the time- and tissue-specific mechanisms of *Shh* regulation (Amano et al., 2009; Williamson et al., 2016; Benabdallah et al., 2019).

Nevertheless, the question remains of how pre-established chromatin changes can be temporally uncoupled from transcription, whilst preserving specificity and robustness of cell-fate decisions. Interestingly, recent papers propose that E-P interactions may be memorised into longer-lived promoter states that integrate past and current signals, suggesting that transcriptional output may vary between cells if they have been exposed to different signals (Xiao et al., 2021; Zuin et al., 2021). Nevertheless, elucidating the mechanisms behind long-lasting promoter memory remains challenging given the transient nature of E-P contacts and the rapid kinetics of proteins and RNA. Alternatively, contact-independent E-P communication has been proposed, a model in which information present at *cis*-regulatory regions is transferred to promoters even at a distance, for example by diffusion of post-translationally modified TFs at enhancers (Karr et al., 2021). Enhancer RNAs or condensates may similarly facilitate transcriptional activation at a distance (Morf et al., 2020). Although contact-independent E-P communication requires greater experimental validation, it offers a theoretical framework compatible with recent findings.

## Discussion

In summary, proximity-based methods and live-imaging approaches are beginning to provide both genome-wide and spatiotemporal information on E-P communication. However, there is no one approach that combines all of this information. As such, physical and computational models of chromatin that integrate datasets collected at different spatiotemporal scales will likely be needed to provide additional insights into the underlying chromatin organisation and regulation. Recent examples of such models reveal that E-P contact frequencies are sensitive to genomic distance, but that promoter activation is likely threshold-dependent (Despang et al., 2019; Yokoshi et al., 2020; Zuin et al., 2021). This translates to a sigmoidal response, with activation responding to a hypersensitive regime around the threshold (Xiao et al., 2021). Interestingly, sigmoidal responses during vertebrate hindbrain segmentation result in a bistable switch regulating cell-fate commitment (Bouchoucha et al., 2013). These findings highlight the importance of using computational models to explore competing hypotheses. Indeed, the prospect of integrating novel single-molecule imaging, next-generation sequencing and chromatin modelling approaches holds great promise for the field (Itoh et al., 2021). In particular, we expect important mechanistic insight to be provided by the new generation of multiscale chromatin models that connect atomistic features of nucleosomes and proteins to the emergence of chromatin organisation and its phase separation (Farr et al., 2021). Integration of simulations and experiments could propose ensembles of E-P configurations with molecular resolution that are both consistent with the experimental data and can be rationalized mechanistically from fundamental physicochemical principles. In addition, since chromatin responds dynamically to extrinsic signals, we expect more tools to reveal E-P communication within a tissue context. Although initial *in vivo* studies have mostly been in zebrafish and *Drosophila*, the growing repertoire of tools will no doubt lead to similar studies within mammalian tissues.
